# Assessing the Spatial Concentration and Temporal Persistence of Poverty: Industrial Structure, Racial/Ethnic Composition, and the Complex Links to Poverty

**DOI:** 10.1007/bf03354897

**Published:** 2015-05-14

**Authors:** Katherine J. Curtis, Perla E. Reyes, Heather A. O’Connell, Jun Zhu

**Affiliations:** a University of Wisconsin-Madison; b Kansas State University

**Keywords:** County poverty, race/ethnicity, industrial structure, Upper Midwest, spatial-temporal autocorrelation, maximum likelihood estimation

## Abstract

This study assesses the social-structural, spatial, and temporal dimensions of aggregate-level poverty in the US Upper Midwest between 1960 and 2000. Central focus is on the links between local-area poverty, industrial structure and racial/ethnic composition, and the spatial and temporal dimensions of the linkages. During the study period, the region underwent significant industrial restructuring and dramatic change in racial/ethnic concentration. Using newly developed statistical methods for spatial-temporal regression, we explore hypotheses related to the spatial and temporal dimensions of the complex relationship between poverty, industrial structure, and race/ethnicity. Our approach yields reliable and interpretable estimates for structural factors of interest as well as the spatial-temporal autocorrelation structure underlying the data. Results inform theory about the implications of industrial structure and racial/ethnic composition for the concentration and persistence of poverty with clear direction for future research, and contribute to our understanding of the methodological approaches to investigating data that varies by and is dependent on space and time.

In spite of having one of the highest average incomes in the industrialized world, the United States has one of the highest poverty rates (Iceland 2006; Smeeding et al. 2001). It is generally accepted that social and economic factors underlie patterns of poverty within the United States. Taken as a whole, previous research has identified industrial structure and racial/ethnic composition as key social-structural contributors to local-area poverty (e.g., Cohn and Fossett 1995; Tickamyer and Tickamyer 1988; Tigges and Tootle 1993; Tomaskovic-Devey and Roscigno 1996; Weingberg 1987). Central to our research, these social and economic factors are time-variant and are spatially differentiated.

Research has explored the temporal patterning of poverty. For instance, poverty declined in recent decades, falling from 13.7% in 1969 to 11.3% in 1999 (Dalaker 2001; U.S. Census Bureau 1993). However, recent data have shown that poverty is on the rise with nearly 48 million Americans (15.9%) living in poverty in 2011 (American Community Survey 2012). Scholars have also recognized a spatial trend in poverty in the United States and the role that place has played in aggravating and perpetuating poverty (e.g., Adams and Duncan 1992; Glasmeier 2006; Lobao and Saenz 2002). Yet studies that analyze both spatial and temporal patterns of poverty are much less common. Exceptions are Jha (2000) who employed descriptive techniques but without statistical inference, and Chokie and Partridge (2008) who included the spatial and temporal effects as fixed rather than dynamic. Simultaneous modeling and evaluation of social-structural factors and spatial-temporal patterning of poverty are largely absent and, yet, are needed to develop a comprehensive understanding of the complex factors that operate in tandem to generate poverty. To date, no study has fully assessed, simultaneously, the spatial and temporal structure of poverty.

Our study takes a first step toward filling this void. The primary research objective is to advance a methodological strategy to more accurately link industrial structure and racial/ethnic concentration to economic vulnerability measured as county-level poverty. Specifically, we aim to produce unbiased estimates that characterize the long-run relationship between poverty and key theoretical social factors. While we have strong theory to guide our analysis, it is a substantial challenge to analyze spatial-temporal data and to draw reliable conclusions. Unlike spatial statistics which have matured over the last several decades, statistics that take into account both space and time are still an active area of research (Cressie 1993; Cressie and Wikle 2011). For instance, adding a temporal dimension to the spatial domain increases modeling complexity. Additionally, the increasing amount of available data often outpaces the increasing speed of modern computers and, thus, requires more efficient computing algorithms. These challenges are not exclusively technical. Rather, they require the researcher to carefully select a modeling strategy that represents efficiently and parsimoniously a complex social event or process.

We extend previous research on place-based poverty that identifies industrial structure and racial/ethnic concentration as key explanations for poverty by explicitly assessing the extent to which the magnitude of the relationships persists after accounting for underlying temporal and spatial dimensions. We do so while assessing the underlying spatial and temporal patterns of poverty by applying new statistical methodology developed for simultaneous model selection and parameter estimation. Our strategy yields a relatively parsimonious model that reflects a complex conceptual model. An essential innovation is to report estimates that account for spatial and temporal autocorrelation, thus permitting us to draw better informed conclusions about poverty and its links to time-variant and spatially distributed structural factors.

Specifically, we examine two hypotheses elaborated in studies of aggregate poverty. First, we test whether a high concentration of less secure industries is positively associated with poverty, net of spatial and temporal autocorrelation. Second, we test whether a high concentration of racial/ethnic minority populations is positively associated with poverty, net of spatial and temporal autocorrelation. Through model selection strategies, we also identify which industries and race/ethnic groups are most strongly associated with poverty. We draw on census panel data for the US Upper Midwest between 1960 and 2000 to test our hypotheses and to apply our modeling strategy. The study region and period provide an ideal site for our research aims given the case’s significant industrial restructuring in the manufacturing and agricultural sectors during this period as well as the marked change in the racial/ethnic composition and associated distribution of the population.

## SPATIAL AND TEMPORAL DIMENSIONS OF POVERTY

A large portion of research on poverty focuses on individuals in contemporary settings. We adopt an alternative view to gain deeper insight into the process underlying aggregate-level poverty, a view rooted in the traditions of historical and spatial demography. While we are not the first to conduct an analysis of aggregate-level poverty in the United States, to our knowledge, we are the first to empirically assess the spatial and temporal dynamics of poverty with the goal of directly testing hypotheses that implicate the spatial and temporal structure. Doing so enables us to better estimate coefficients in terms of accuracy and, consequently, to gain a better understanding of aggregate poverty.

Within poverty research, scholars have recognized that poverty is spatially patterned and that attributes of place contribute to promoting and even reproducing poverty (Adams and Duncan, 1992; Lobao and Saenz, 2002; Lobao, 2004; Glasmeier, 2006). Researchers have given varying degrees of analytical attention to Appalachia, the Mississippi Delta, the southwestern borderlands, and tribal reservations and communities (Billings and Blee, 2000; Dill and Williams, 1992; Duncan, 1992; Snipp and Summers, 1992; Poston et al., 2010; Voss et al., 2006). Moreover, researchers have conceptualized place (e.g., Lichter et al., 1993; Lichter and McLaughlin, 1995; Cotter, 2002), analyzed poverty within spatial units (e.g., Lobao, 1990; Friedman and Lichter, 1998; O’Hare and Johnson, 2004), examined short-term concentrated poverty (Lichter and Johnson, 2007), and explicated the historical underpinnings of poverty in select sub-regions (e.g., Billings and Blee, 2000; Dill and Williams, 1992; Duncan, 1992; Snipp and Summers, 1992). This body of research has demonstrated that poverty and the factors promoting poverty are unevenly distributed across the United States (McLaughlin and Perman, 1991; Lichter et al., 1994; Friedman and Lichter, 1998; Curtis et al., 2012). For example, places with greater dependence on a single, contracting industry tend to have higher proportions of residents living in poverty. Industrial structure is not uniformly distributed across space; thus, pockets of higher and lower poverty emerge.

Research has also demonstrated that high-poverty counties, for the most part, have been impoverished for decades (Beale and Gibbs, 2006). We examine whether persistence in poverty corresponds with stability in the theorized dominant factors of poverty, most especially industry. Dramatic industrial shifts (i.e., deindustrialization) have been shown to reorient local and regional economic development (Kasarda 1989, 1995; McCall 2001). Thus, trends in poverty might reasonably follow trends in industrial expansion or contraction. Consequently, places with strong ties to industries that are vulnerable to contraction are also vulnerable to increases in poverty. Alternatively, the relationship between an industry and poverty might weaken or reverse over time due to changes within the industry itself. As a result, the effect of industry over the period might be washed out. Moreover, research has shown that the impact of local labor market conditions are mediated in some periods by social factors, including family structure and racial/ethnic composition (Snipp and Summers, 1992; Saenz, 1997; Friedman and Lichter, 1998; Green and Sanchez, 2007), as the relationship between industry and social factors changes over time (e.g., feminization of certain industries and racial segregation among industries).

Poverty is generally understood to be influenced by multiple social and economic factors. In this study, we focus on poverty in relation to racial/ethnic composition as a key social factor and industrial sector as a key economic indicator. The two factors are linked to one another and we take the position that racial/ethnic composition is not a cause of poverty. Instead, high racial/ethnic concentration might be positively correlated with poverty given exploitative and discriminatory practices that are more deeply rooted or widely practiced within certain industries (Snipp, 1996; Saenz, 1997). Both industries and racial/ethnic groups are unevenly distributed across space and, for some sectors and groups, over time.

While not equivalent to poverty, the role of regional structure and spatial interactions is a growing focus in the regional science literature (Oosterhaven et al., 2001; Anselin et al., 2004; Ramajo et al., 2008; Rey and Sastre-Gutierrez, 2010). Research has demonstrated that regional development is spatially patterned, though the spatial structure is not uniform across sub-regions (i.e., states) within the larger geography (i.e., nations). That is, development is spatially heterogeneous and spatially concentrated. Moreover, recent efforts have examined the temporal dimension of space in terms of macro-economic shifts shaping the evolution of regional inequality (Rey and Montouri, 1999; Fingleton, 2004; Rey, 2004; Fingleton and Lopez-Bazo, 2006; Rey and Le Gallo, 2009; Rey and Sastre-Gutierrez, 2010).

Our approach is complementary to this body of literature in suspecting that spatial and temporal processes generate the unequal distribution of county poverty in the United States. However, our approach is distinct in two ways. First, substantively, the regional science literature generally focuses solely on the economic dimension (i.e., income growth), whereas our approach focuses on the economic dimension in relation to the social dimension. Second, technically, the literature jointly treating spatial and temporal dynamics generally uses advanced spatial analytic tools to characterize spatial-temporal patterns, whereas our explicit aim is to advance statistical methods of data analysis that relate the spatial-temporal pattern to possible explanatory factors; our analysis accounts for the spatial-temporal pattern and, at the same time, quantifies the relationship between poverty and key social factors.

Embedded in discussions of economic vulnerability is the complex system of structural (or attribute), spatial and temporal dimensions which underlie different rates of poverty. Previous research has established that poverty and the key structural factors associated with it are unevenly distributed across space. Similarly, temporal trends in poverty are presumably paralleled by trends in key structural predictors. These substantively meaningful associations might be confounded by underlying endogenous processes, namely autocorrelation. Concerns about appropriate estimates stressed in time series analysis and spatial data analysis are still at play, indeed perhaps they are magnified in the context of spatial-temporal analysis. That is, the temporal and spatial autocorrelation structure must be accounted for in order to produce consistent and efficient estimates of key predictors. Failure to account for the autocorrelation structure will likely produce suboptimal parameters, which may ultimately mislead theoretical conclusions. As a first step in accounting for the spatial and temporal dimensions of poverty, we extend previous research on place-based poverty that identifies industrial structure and racial/ethnic concentration as central explanatory variables of poverty by explicitly assessing the extent to which the relationships persists after accounting for spatial and temporal autocorrelation.

### The Midwestern Case

Our analysis of the Upper Midwestern United States is comprised of Illinois, Indiana, Michigan, Minnesota, and Wisconsin. The region has experienced diverse economic and racial/ethnic trends since the 1960s. Thus, the Upper Midwest provides an ideal case to examine the theorized relationships of concern. Deindustrialization occurred during the 1980s and most heavily impacted manufacturing as once secure and well-paying manufacturing jobs moved southward within and beyond the US borders (Kasarda, 1989; 1995). At that time, manufacturing was largely concentrated in the eastern-most states comprising the region, especially in counties making up the central part of the region. Correspondingly, industry contractions were also concentrated in these areas, presumably with implications for poverty. By comparison, the more westward states and counties comprising the northernmost and southernmost parts of the region were more heavily dependent on agriculture, including forestry. The agricultural sector experienced its share of economic contraction in the 1980s also with implications for poverty. Importantly, within the Upper Midwest, the agriculture and manufacturing sectors have been historically tightly linked (White, 2008); agricultural goods produced in the region are largely processed in the region. Therefore, the full potential for economic contraction within the manufacturing sector might be lessened by its interdependence with agriculture. A third, emerging industry in the Upper Midwest during the study period is the service sector. Growth in this sector, however, might not necessarily reduce economic vulnerability given the less secure qualities that tend to characterize service jobs (e.g., low pay, low benefits, and low hours) (Collins and Mayer, 2010).

We also know that racial/ethnic groups are not equally represented in all industries or in all places within the Upper Midwest. Large numbers of African Americans settled in the more urbanized states and in the more metropolitan areas within these states during prosperous historical periods of wartime industrial expansion (Henri, 1975; Tolnay et al., 2002; Gregory, 2005). Consequently, a large population of African Americans was employed in manufacturing and living in metropolitan areas at the time of deindustrialization. At the same time, employment among the Hispanic population has been historically concentrated in the agricultural sector, although increasingly employed in manufacturing in recent decades. Manufacturing within the Upper Midwest was once largely centered on durable goods and later shifted to the processing of agricultural goods which relied on a large Hispanic labor force (Kandel and Parrado, 2005). Due to the comparatively high rates of population growth, through in-migration and fertility, the Hispanic population has become increasingly represented in metropolitan and non-metropolitan areas over time (Donato et al., 2007; Liaw and Frey, 2007; Lichter and Johnson, 2009). Concurrently, patterns of residential segregation between African Americans and whites were heightened by white flight to suburbs (Massey and Denton, 1993), often spilling over county boundaries. As the population became increasingly racially sorted, so too did industry (Wilson, 1996); professional jobs tended to follow whites whereas economic growth in areas with large minority populations centered on the more casualized and less secure service sector (Collins and Mayer, 2010).

Taken together, trends in key economic and social factors within the Upper Midwest make it a compelling case to explore the spatial and temporal dimensions of poverty. In the following section, we describe our analytical strategy for simultaneously assessing the structural factors that contribute to poverty and the accompanying autocorrelation structure to yield a more comprehensive test of long-standing hypotheses about aggregate-level poverty.

## DATA AND MEASURES

**Figure 1.**
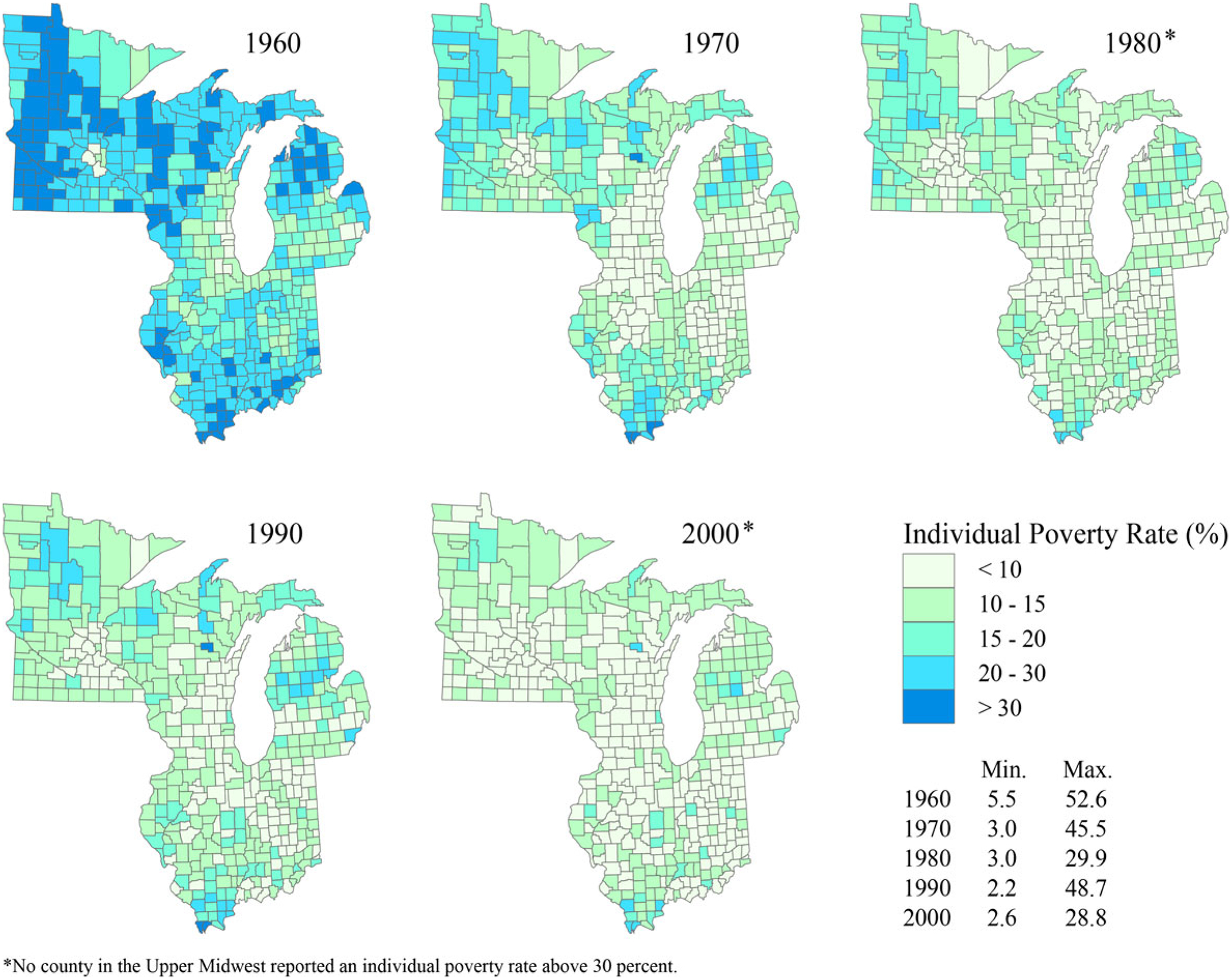
Decade-specific county poverty rates with minimum and maximum values. Upper Midwest counties, 1960–2000.

We apply newly developed spatial-temporal regression techniques, which we elaborate in the next section, to analyze county-level poverty rates between 1960 and 2000 in five states in the Upper Midwest: Illinois, Indiana, Michigan, Minnesota, and Wisconsin. The data are drawn primarily from the Census of Population (1960, 1970, 1980, 1990, and 2000). Our dependent variable is the reported poverty rate, based on the proportion of the county population living below the poverty threshold. The measure is ideal for our purposes since it is comparable across time. Figure 1 shows the poverty rates by county in the five Midwestern states from the 1960 census through 2000. The severity of poverty was not stable across the study period. Poverty was highest in 1960, with the majority of counties in the upper Midwest reporting poverty rates at 10% or higher. Poverty declined most dramatically in the 1960s during the nation’s War on Poverty policy effort. Rates continued to decrease through the 1970s, rebounded during the 1980s, and returned to a decline in the 1990s. Although the extent of poverty varied over this period, the relative spatial distribution of poverty was stable. The value of the highest poverty rate was not the same across the period, yet the highest rates were consistently concentrated in the northernmost and southernmost counties of the upper Midwest. Similarly, the lowest levels of poverty fell within the mid-section of the region. Overall, as poverty generally declined between 1960 and 2000, pockets of concentrated poverty contracted, leaving increasingly fewer counties at a growing disadvantage relative to other counties within the region.

Our analytical focus is on poverty in relation to two structural factors, industrial structure and racial/ethnic composition. Local-area industrial structure is represented by variables reflecting the proportion of the civilian population 16 years and older per county that is employed in dominant industries including agriculture, manufacturing, services, mining, FIRE (finance, insurance, and real estate), and other professionals (e.g., science, technology, education).^1^ In our analysis, manufacturing, agriculture, and service are of central interest while measures of the other industries are of secondary substantive concern. An area’s racial/ethnic composition is represented by several variables reflecting the proportionate size of dominant racial/ethnic minority groups in the United States and in the Upper Midwest including the African American, American Indian, and Hispanic population relative to the total county population, as well as the proportionate size of the non-Hispanic white population. Analytical attention is limited to these two factors in our initial foray, although we anticipate investigating the role of other factors, most centrally family structure, in future work. We are motivated to present the most parsimonious model given the statistical complexity and computational demands of integrating the spatial-temporal autocorrelation structure. Our choice of factors is justified given the emphasis on industry and race/ethnicity in aggregate-level studies of poverty (e.g., Cohn and Fossett, 1995; Tickamyer and Tickamyer, 1988; Tigges and Tootle, 1993; Tomaskovic-Devey and Roscigno, 1996; Weingberg, 1987).

We analyze county level poverty for two reasons. First, we are interested in investigating the association between county-level factors and county poverty. This research is not concerned with individual-level processes that contribute to poverty; instead, we are interested in understanding the spatial inequality in economic vulnerability among counties. Second, the county is a sensible unit of interest because it embodies the structural factors that produce economic vulnerability. Poverty happens to communities and to places. From a social demographic perspective, counties are geographic units that represent socially constructed yet physically bounded areas that hold characteristics which interact with location to create divergent social, economic, and political outcomes.

## ANALYTICAL CHALLENGES AND APPROACH

Statistical methods based on probabilistic modeling, in theory, provide rigorous approaches to analyzing our data and testing our general hypotheses. For example, spatial-temporal regression helps identify important drivers of poverty rates. However, it remains a challenge in practice to carry out spatial-temporal data analysis because spatial-temporal statistics that take into account both space and time are still at a somewhat early stage of development. This lag in development is possibly due to the increased level of model complexity. Although it has been an active area of research, modeling and inference are generally based on the Bayesian hierarchical modeling framework (Banerjee et al., 2004). While flexible and powerful, Bayesian inference tends to be computationally intensive (Zheng and Zhu, 2008) and model selection is not always adequately addressed for spatial-temporal modeling in part because of high computational costs. We take an alternative, maximum likelihood based approach that is statistically rigorous, performs systematic model selection and permits efficient computational algorithms that enable practical use. This approach is more computationally intensive than standard models that do not account for underlying autocorrelation or select on variables. However, we assert, and demonstrate, that the additional cost in computational time is worth the investment since our approach yields a parsimonious theoretical model with consistent and efficient estimates.

Variable selection via penalized methods for standard linear regression has been an active area of research in the last decade or so. Innovations include least absolute shrinkage and selection operator (Lasso) (Tibshirani, 1996), adaptive Lasso (Zou, 2006), and penalized least squares or maximum likelihood under non-concave penalty (Fan and Li, 2001). While most penalized methods assume independence, results for dependent data are emerging, for example, for time-series data (Wang et al., 2007) and spatial data (Huang et al., 2010; Zhu and Liu, 2010; Zhu et al., 2010). Here, we use a recently developed methodology that permits us to analyze spatial-temporal models with simultaneous selection of covariates and spatial-temporal autocorrelation structures (Reyes, 2010). These methods have sound asymptotic properties including consistency, sparsity and asymptotic normality.

The spatial-temporal regression model features regression on the structural factors of substantive concern, spatial autocorrelation, temporal autocorrelation, and spatial-temporal interactions. Specifically, for county *i* and time *t*, let *y*_*i,t*_ = *β*_*0*_ + *β*_*1*_*x*_*1,i,t*_ + *β*_*2*_*x*_*2,i,t*_ +*…+ β*_*p*_*x*_*p,i,t*_ + *ε*_*i,t*_, where the response variable is *y*_*i,t*_, the explanatory variables are *x*_*1,i,t*_, *x*_*2,i,t*_, *…, x*_*p,i,t*_, and the error process ε_i,t_ is modeled by a Gaussian process. The regression coefficients *β*_*0*_, *β*_*1*_, *β*_*2*_, *…, β*_*p*_ are unknown parameters. For the error process, the approach is similar to a simultaneous autoregressive model for spatial-only data. In particular, let ***ε***_*t*_ = ***C***_*0*_***ε***_*t*_ + ***C***_*1*_***ε***_*t-1*_ + *…+* ***C***_*L*_***ε***_*t-L*_+ ***v***_*t*_, where the vector ***ε***_*t*_ consists of the errors at all counties and time *t*, ***v***_*t*_ are assumed to be iid Gaussian variables with constant variance σ^2^, and ***C***_*l*_ are matrices of autoregressive coefficients among spatial-temporal neighbors. When *l=0*, ***C***_*0*_ is for the autocorrelation among spatial neighbors at the same time point. For *l=1,2,…,L* the matrix ***C***_*l*_ is for the autocorrelation among spatial neighbors but with l time lag apart. These autoregressive coefficient matrices ***C***_*l*_’s are parameterized by a vector of unknown spatial-temporal coefficients denoted as ***Θ***.

Let ***y*** denote the vector of response variables (poverty rates) and ***X*** denote the design matrix that comprises the factors (industry and race/ethnicity). The response vector ***y*** therefore follows a multivariate normal distribution with a mean vector ***Xβ*** and a covariance matrix ***Γ***, which is essentially a spatial linear model. The log-likelihood function for the model parameters ***β***, **Θ**, and σ^2^ is

$$\text{Log-likelihood}\;\text{function}=-(1/2)\text{ln}|\Gamma (\Theta ,\sigma^{2})|-(1/2)(y-X\beta )’\Gamma {\left(\Theta ,\sigma^{2}\right)}^{-1}(y-X\beta ).$$

For variable selection, a penalized log-likelihood function can be constructed as the negative log-likelihood function plus a penalty function for ***β*** and ***Θ***. The particular penalty function used here is known as adaptive Lasso (see Reyes, 2010). The values of ***β*** and ***Θ*** that maximize the penalized log-likelihood function are the penalized maximum likelihood estimates (PMLE). Under PMLE, some of the regression coefficients and spatial-temporal coefficients are shrunk to zero, and thus the PMLE approach can be used for selecting both covariates and spatial-temporal dependence. When a particular regression coefficient is estimated to be zero, then the corresponding covariate will not be included in the selected model. Similarly, via the selection of spatial-temporal coefficients, the spatial-temporal autoregressive structure can be determined.

In our analysis, the spatial covariance structure follows the simultaneous autoregressive model described above with a flexible class of parameterizations that features spatial-temporal interactions. This approach enables us to examine the substantive links between local area poverty and industry and racial/ethnic composition while simultaneously accounting for the spatial and temporal processes not captured by the structural factors. Through this approach, we are able to report estimates of the links between structural factors and poverty purged of bias or inefficiency introduced by autocorrelation in the data and, related, draw informed theoretical conclusions about the associations between industrial structure, racial/ethnic composition, and poverty. As with previous spatial studies of county poverty, we apply a first-order queen continuity weight matrix to address spatial autocorrelation (Curtis et al. 2012; Voss et al. 2006).

In addition, our modeling strategy permits us to draw substantive conclusions about the spatial and temporal dimensions underlying poverty. In terms of the spatial dimension, we report a spatial association that is separate from the temporal process; thereby permitting us to advance our knowledge about the role of space in poverty. In terms of the temporal dimension, we report a temporal association that is separate from the spatial process; thereby permitting us to speak to the persistence or “stickiness” of local-area poverty. We also report a temporally-lagged spatial association, which captures the interdependence of the two dimensions; thereby permitting us to speak to the interaction of the two autocorrelated processes.

Three modeling strategies are considered in our analysis. In the first case (I), the covariates and the spatial-temporal autocorrelation structure are selected simultaneously using penalized maximum likelihood estimation (PMLE) described above. In the second case (II), the spatial-temporal autocorrelation structure is included, but no model selection is performed and the results are simply maximum likelihood estimates (MLE). In the third case (III), a standard ordinary least squares (OLS) regression is fit (i.e., covariates are not selected and we do not account for underlying spatial-temporal autocorrelation). The comparison of models is intended to demonstrate the benefits of our preferred modeling strategy with regard to accounting for spatial-temporal autocorrelation (cases I and II versus case III) and variance and model selection (case I versus case II).^2^

## RESULTS

The estimates and standard deviations for the three cases are presented in Table 1. In terms of model selection, we observe that when the structural factors are selected (case I) the proportion white is the only race/ethnic group that is selected, whereas the proportion employed in mining is the only industrial sector that is eliminated. When neither the spatial-temporal autocorrelation structure nor the covariates is selected (case II), the Bayesian Information Criterion (BIC) value increased and is thus worse in terms of model fitting and parsimony. Moreover, the PMLE and MLE models (cases I and II) outperform the OLS model (case III) in terms of fitting as indicated by the smaller BIC values.

**Figure 2.**
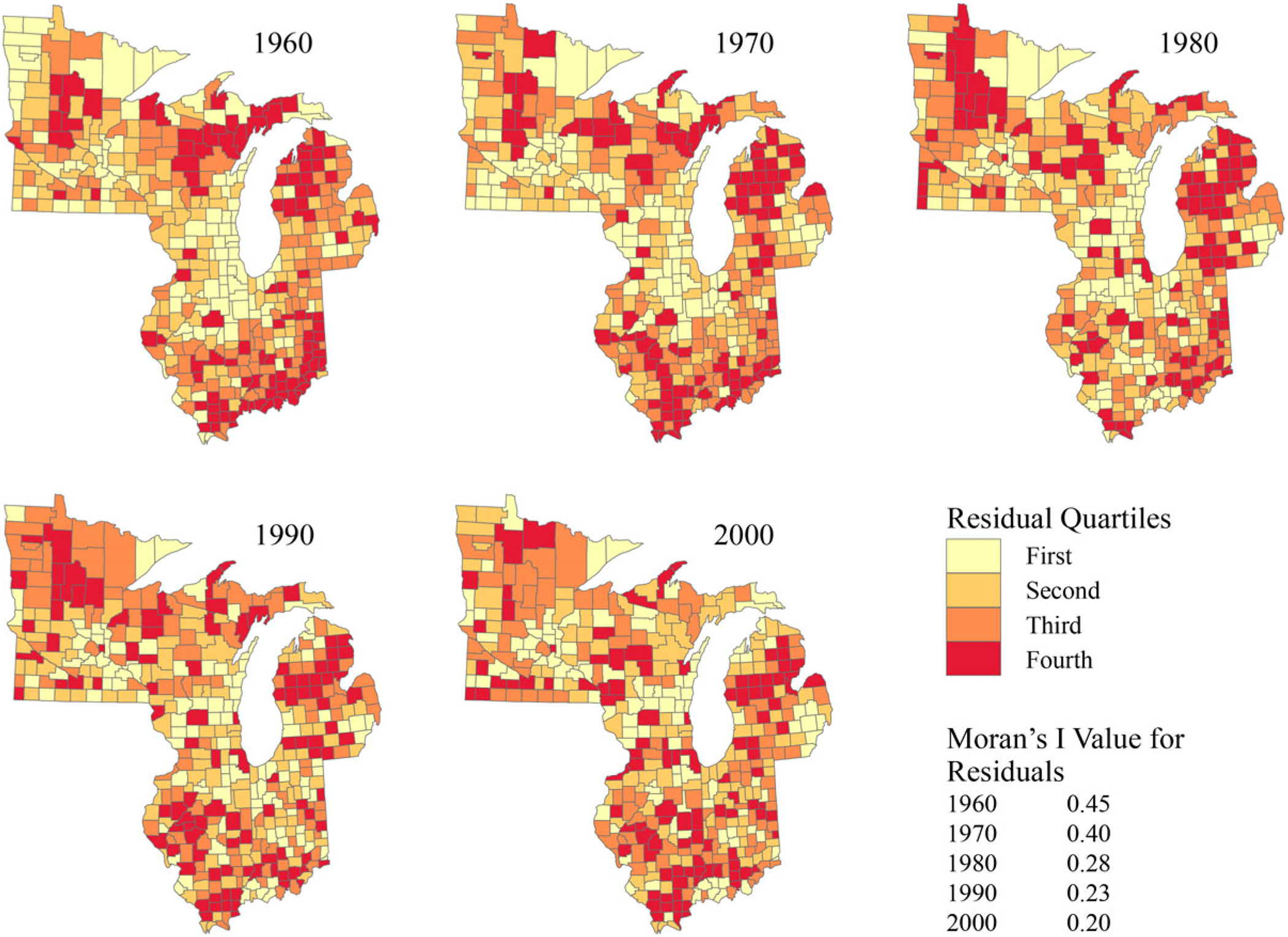
Spatial distribution of decade-specific residuals from standard linear regression analysis (OLS) of county poverty with Moran’s I statistics for spatial autocorrelation (p-value < .001 in each decade). Upper Midwest counties, 1960–2000.

Two general conclusions can be drawn from the results: there is evidence that the spatial-temporal autocorrelation structure of poverty is complex; and not all social or economic factors are needed to explain the variability of county-level poverty. Our first claim is supported by the lower BIC values for the space-time interaction models (case I and II) compared to the standard linear regression model (case III) which assumes no underlying autocorrelation structure (OLS BIC=1509.93 versus PMLE BIC=−516.87 and MLE BIC=−508.63). This implies that it is beneficial to consider spatial and temporal autocorrelation in order to obtain more accurate and precise parameter estimates for the structural factors of substantive interest. A review of the residuals from the OLS model adds further support for the models that account for spatial-temporal autocorrelation. Figure 2 shows the extent and pattern of spatial autocorrelation that persists after accounting for the structural factors through an OLS strategy. We have presented the residuals as quartiles because the specific values of the residuals vary across decades. Negative residuals are captured in the lower quartiles whereas positive values fall within the higher quartiles. Moran’s I statistics, calculated from the OLS residuals by decade and reported in the bottom-right corner of Figure 2, suggest that poverty was positively spatially autocorrelated in the Upper Midwest although the magnitude of the correlation steadily diminished over the study period (Moran 1950). Ultimately, had we not autocorrelation in addition to the temporal autocorrelation, the quality of the estimated coefficients for the key structural factors of poverty would have been compromised. For instance, in comparing cases II and III, the sign of the coefficient for mining reverses after accounting for spatial-temporal autocorrelation. Although the OLS results suggest that mining is positively associated with poverty in the Midwest, we find that it is negatively associated after accounting for the underlying spatial-temporal autocorrelation structure. Moreover, nearly every coefficient is different—sometimes larger, sometimes smaller—in the spatial-temporal regression results as compared to the coefficients estimated through an OLS.

Results also show that after considering the structural factors, the nature of the residual autocorrelation structure is complex; there is a temporal dimension of poverty that is separate from the spatial dimension, as well as an interaction between the two dimensions. This space-time autocorrelation structure is stable, as the full structure was preserved after performing selection (case I). The direction of the spatial and temporal parameters gives some insight into the nature of the interplay between the dimensions. The spatial parameter for the current period is positive, suggesting that neighboring counties have similar poverty rates; thus supporting the claim that poverty is concentrated in space. Similarly, the positive temporal parameter indicates that a county has a similar poverty rate in the current period as in the previous period; thus supporting the claim that poverty is persistent over time. Considering the two dimensions together, reported as the spatial parameter for the previous period, the negative coefficient suggests that the spatial concentration is less stable over time, and the temporal persistence is less enduring across space. Otherwise stated, a county’s current poverty rate has a negative association with its neighbors’ previous poverty rate. This finding is consistent with the spatial and temporal patterns reported in Figure 1. There is some variation in poverty across space and over time, implying that while there is no radical change, not all places are equally vulnerable and no place is equally vulnerable in all decades. Poverty is dynamic.

The second main conclusion drawn from the analysis is that only a subset of the tested social and economic factors is required to model poverty as revealed in the case were the model is selecting on the structural covariates (case I). The proportions of the dominant minorities are no longer significant factors after the proportion white is considered. A review of cases II and III shows that the racial/ethnic composition and industrial sector variables are generally associated with poverty in the anticipated direction. The estimated parameters for racial/ethnic composition are negative for the proportion white and Hispanic, but positive for African American and American Indian. This indicates that counties with a larger proportion of African American and American Indian tend to have a higher poverty rate, whereas counties with a larger concentration of whites and Hispanics tend to have a lower poverty rate.

However, when residual spatial-temporal structure is considered and variables are selected, only white concentration maintains a statistically significant association with poverty, suggesting that other structural factors—perhaps white concentration or industrial sector—or underlying spatial-temporal trends account for the relationship between poverty and racial/ethnic groups of color.

Results from case I also show that nearly all of the industrial sectors are significantly associated with poverty with the exception of mining. These results support the claim that industrial structure plays a key role in shaping county poverty. However, the various sectors are related to poverty in different ways. The estimated regression coefficients for the industrial sector are negative for manufacturing, finance/insurance/real-estate (FIRE), and other professions; counties with higher concentrations of these industries tend to have lower poverty. In contrast, the coefficients for agriculture and service are positive, indicating that counties with higher concentrations of these sectors tend to have higher poverty. The findings for the agriculture and service sectors are consistent with our *a priori* expectations; places with high concentration of these industries are more economically vulnerable likely due to low pay and low benefits. In contrast, poverty appears to be lower in places with higher employment in manufacturing.

## DISCUSSION

Our method permits us to fit a comprehensive model of the structural, temporal and spatial dimensions of poverty. Consistent with previous research, we find that poverty is linked to industrial structure and racial/ethnic composition, factors that are linked to economic vulnerability and are embedded in the broader social and economic dimensions facing the Upper Midwest during the study period. However, unlike earlier studies, we explicitly parameterize the entangled spatial and temporal associations underlying county poverty. As a result, we produce consistent and efficient estimates of the structural factors while gaining insight on the underlying spatial-temporal autocorrelation structure.

Midwestern counties with higher concentrations of employment in agriculture and service industries tend to report higher poverty rates than other counties. These industries are less secure in terms of wages and other worker conditions (e.g., Collins and Mayer, 2010), putting places dependent on these industries at a relative disadvantage. These patterns persist even after accounting for spatial-temporal autocorrelation. We also show that counties with higher concentrations of non-Hispanic whites had a higher probability of reporting lower poverty during the period as compared to counties with relatively larger proportions of populations of color. The relative disadvantage of counties with concentrations of racial/ethnic minorities within the Midwest is consistent with arguments about economic underdevelopment on reservations and within dense urban areas, with important implications for the racial and ethnic face of poverty (Wilson, 1987; Snipp, 1996). However, our results demonstrate that the relationship between concentrations of populations of color and poverty are tenuous; no association is found when selecting on structural covariates (racial/ethnic composition and industrial structure). The only race/ethnic group persistently associated with poverty is the non-Hispanic white population. Places with high concentrations of whites tend to have lower poverty independent of economic factors and spatial-temporal trends.

Our approach also provides empirical evidence for simultaneous spatial and temporal associations. Counties tended to neighbor counties with similar poverty rates, and counties tended to report a similar level of poverty throughout the study period. Findings are consistent with previous observations about the temporal persistence of poverty and the spatial concentration of poverty (e.g., Jargowsky, 1997; Glasmeier, 2006; Lichter and Johnson, 2007). A significant difference between our approach and those taken in previous studies is our ability to simultaneously address the spatial and the temporal associations by explicitly parameterizing them, as well as parameterizing a second-order association between the two dimensions. The contribution is more accurate coefficients for the structural factors, as well as a more comprehensive understanding of how the spatial and temporal dimensions of poverty operate.

While we focus on the Upper Midwest, our strategy can be applied to the entire United States or generalized to any other geography. It can also be extended to estimate region-specific models in order to test theoretically-based assertions of distinct place-based processes (e.g., Curtis et al., 2012; McCall, 2001) or temporally distinct regimes (e.g., Messner et al., 1999). Future research could expand the study region to include other Midwestern states, thereby bolstering the ability to address industry-specific shifts in the relationship to poverty over time. Additionally, incorporating the most recent decade, that of the Great Recession, would enable researchers to assess the extent to which our results persist in extreme macro-economic conditions. Such work could investigate suspected temporal regimes that correspond to macro-economic shifts (i.e., deindustrialization, recession) by disaggregating the temporal patterns. Moreover, future research could include additional covariates to further probe the social and economic factors underlying the spatial concentration and temporal persistence of poverty.

## Figures and Tables

**Table 1.** Estimated regression coefficients (β) and standard deviation (SD) of model parameters for (I) nonzero approximate penalized maximum likelihood estimate with selection of both covariates and spatial-temporal autocorrelation structure; (II) maximum likelihood estimate with spatial-temporal autocorrelation structure; and (III) standard OLS with no selection. Upper Midwestern counties, 1960–2000.

Case	I		II		III	
	β	SD	β	SD	β	SD
Covariates						
White (%)	−0.158	0.027	−0.128	0.027	−0.08	0.041
African American (%)	-	-	0.039	0.023	0.098	0.032
Hispanic (%)	-	-	−0.015	0.011	−0.073	0.015
American Indian (%)	-	-	0.017	0.013	0.015	0.016
Agriculture (%)	0.212	0.012	0.202	0.012	0.078	0.013
Mining (%)	-	-	−0.014	0.007	0.011	0.009
Manufacturing (%)	−0.058	0.012	−0.075	0.012	−0.267	0.013
Service (%)	0.041	0.013	0.051	0.013	0.041	0.009
FIRE (%)	−0.039	0.008	−0.049	0.008	−0.256	0.013
Other professions (%)	−0.016	0.014	−0.047	0.014	−0.23	0.009
Spatial-temporal autocorrelation						
Spatial, current period	0.843	0.012	0.843	0.012	-	-
Spatial, previous period	−0.672	0.026	−0.672	0.026	-	-
Temporal	0.771	0.019	0.771	0.019	-	-
Variance	0.035	0.001	0.035	0.001	0.113	0.337
BIC	−516.87		−508.63		1509.93	

## Footnotes

1 We do not include a control for metropolitan status because research examining industrial structure and rural economic disadvantage asserts that “[r]ural poverty has always been linked to the limited opportunity structure in rural communities” (Tickamyer and Duncan, 1990:81). Our measure of industrial composition accounts for the factor that most significantly drives differences in nonmetro/metro county poverty. Additionally, research suggests that a dichotomous distinction (i.e., nonmetro/metro) does not adequately capture the range of urbanicity that is relevant to economic outcomes (e.g., Wang et al., 2012). We are limited to a dichotomous measure given the timespan under study. Therefore, our industry measure more adequately captures nuanced differences along the urbanicity continuum than would the available dichotomous variable.

2 The parameter estimates in PMLE are obtained from maximizing a penalized likelihood function. In particular, the maximization is solved using a least angle regression (LARS) algorithm featured in the package lars in R (Efron et al. 2004; R Foundation). See Reyes (2010) for a detailed presentation of the method and the underlying statistical theory.
